# Disruption of Rhino Demography by Poachers May Lead to Population Declines in Kruger National Park, South Africa

**DOI:** 10.1371/journal.pone.0127783

**Published:** 2015-06-29

**Authors:** Sam M. Ferreira, Cathy Greaver, Grant A. Knight, Mike H. Knight, Izak P. J. Smit, Danie Pienaar

**Affiliations:** 1 Scientific Services, SANParks, Skukuza, South Africa; 2 School of Biological and Conservation Sciences, University of KwaZulu-Natal, Pietermaritzburg, South Africa; 3 Aerial Support Services, SANParks, Skukuza, South Africa; 4 Park Planning and Development Division, SANParks, Port Elizabeth, South Africa; University of Queensland, AUSTRALIA

## Abstract

The onslaught on the World’s rhinoceroses continues despite numerous initiatives aimed at curbing it. When losses due to poaching exceed birth rates, declining rhino populations result. We used previously published estimates and growth rates for black rhinos (2008) and white rhinos (2010) together with known poaching trends at the time to predict population sizes and poaching rates in Kruger National Park, South Africa for 2013. Kruger is a stronghold for the south-eastern black rhino and southern white rhino. Counting rhinos on 878 blocks 3x3 km in size using helicopters, estimating availability bias and collating observer and detectability biases allowed estimates using the Jolly’s estimator. The exponential escalation in number of rhinos poached per day appears to have slowed. The black rhino estimate of 414 individuals (95% confidence interval: 343-487) was lower than the predicted 835 individuals (95% CI: 754-956). The white rhino estimate of 8,968 individuals (95% CI: 8,394-9,564) overlapped with the predicted 9,417 individuals (95% CI: 7,698-11,183). Density- and rainfall-dependent responses in birth- and death rates of white rhinos provide opportunities to offset anticipated poaching effects through removals of rhinos from high density areas to increase birth and survival rates. Biological management of rhinos, however, need complimentary management of the poaching threat as present poaching trends predict detectable declines in white rhino abundances by 2018. Strategic responses such as anti-poaching that protect supply from illegal harvesting, reducing demand, and increasing supply commonly require crime network disruption as a first step complimented by providing options for alternative economies in areas abutting protected areas.

## Introduction

Mega-herbivores are key species that influence several objectives that conservation agencies seek to achieve [1]. Kruger National Park (Kruger) is a stronghold of several mega-herbivore species including the southern white rhinoceros (*Ceratotherium simum simum*) and the south-eastern black rhinoceros (*Diceros bicornis minor*) [2]. It is here where both species are suffering a poaching onslaught for their priced horns [3]. Responses of authorities focus on managing the threats [4] that poaching poses to the persistence of rhinos, a key measure of which is population size and growth rates.

During 2008, SANParks estimated that 627 (95% CI: 588–666) black rhinos resided in Kruger increasing at 6.75% per annum [5]. During 2010, SANParks estimated 10,621 (95% CI: 8,767–12,682) white rhinos [6]. The Kruger white rhino population is a keystone conservation entity [2] and provided conservation-based revenue historically for SANParks through live sales [7]. Restoring ecological process [8], or if not possible mimicking the outcomes of those processes provide opportunities to reconcile biodiversity and financial objectives [6], with poaching carrying consequences for these values. After becoming extinct in Kruger in 1896 and reintroduced in the 1960’s [9], white rhino numbers increased until 2008. The management effect (*i*.*e*. mimicking population regulation through removal of rhinos for live sales, [6]) on the population contributed together with poaching to the non-growth of the white rhino population after 2008.

Using a combination of black rhino demographic information obtained during 2008 [5] together with recorded poaching incidences between 2008 and 2012, we aim to predict the expected number of black rhinos killed per day as well as their population size during 2013. For white rhinos we use the predictions derived from demographic and poaching trends identified up to 2010 [6]. We first predicted the expected number of rhinos killed per day if trends in poaching rates continue, and second the expected population size of white rhinos during 2013. We check these predictions by evaluating information from an intensive black rhino and white rhino aerial survey during 2013. Poaching reduces abundance [2], but is also likely to disrupt demographic responses of populations to environmental conditions and density-dependent processes as poaching acts as a non-random process (*e*.*g*. bias towards adults) [6]. We thus also extracted demographic profiles for white rhinos and check for potential demographic poaching effects.

## Material and Methods

Permission to conduct the study was granted by SANParks Conservation Management Division of Kruger National Park, South Africa. No animals were handled in the study.

### Study area

**Fig 1:**
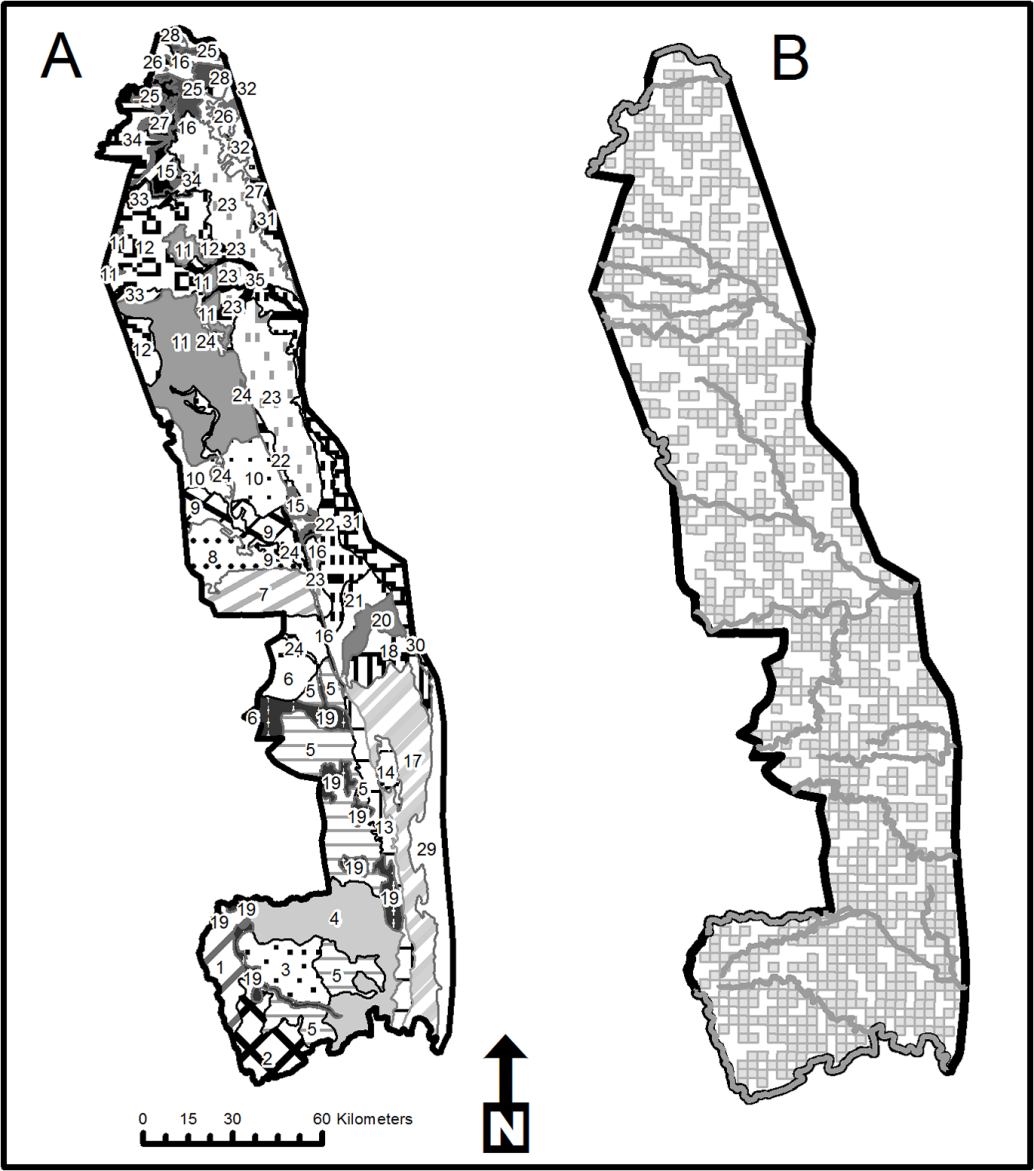
Details of the study area at Kruger National Park. Landscapes within Kruger National Park (Gertenbach 1983) (A) and the location of sample blocks (B) surveyed during 2013. 1. Lowveld Sour Busveld of Pretoriuskop. 2. Malelane Mountain Bushveld. 3. *Combretum colinum*/*Combretum zeyheri* woodland. 4. Thickets of the Sabie and Crocodile River. 5. Mixed *Combretum*/*Terminalia* sericea woodland. 6. *Combretum*/*Colophospermum mopane* woodland of Timbavati. 7. Olifants River Rugged Veld. 8. Phalaborwa Sandveld. 9. *Colophospermum mopane* woodland/savannah on basic soil. 10. Letabe River Rugged Veld. 11. Tsende Sandveld. 12. *Colophospermum mopane*/*Acacia nigrescens* savannah. 13. *Acacia welwitchi* thickets on Karoo sediment. 14. Kumana Sandveld. 15. *Colophospermum mopane* forest. 16. Punda Maria Sandveld on Cave Sandstone. 17. *Sclerocarya birrea* subspecies *caffra*/*Acacia nigrescens* svanna. 18. Dwarf *Acacia nigrescens* savannah. 19. Thornveld on gabbro. 20. Bangu Rugged Veld. 21. *Combretum*/*Acacia nigrescens* rugged veld. 22. *Combretum*/*Colophospermum mopane* Rugged Veld. 23. *Colophospermum mopane* shrubveld on basalt. 24. *Colophospermum mopane* shrubveld on gabbro. 25. *Adansonia digitata*/ *Colophospermum mopane* Rugged Veld. 26. *Colophospermum mopane* shrubveld on calcrete. 27. Mixed *Combretum*/*Colophospermum mopane* woodland. 28. Limpopo/Livhuvhu Floodplains. 29. Lebombo South. 30. Pumbe Sandveld. 31. Lebombo North. 32. Nwambiya Sandveld. 33. *Pterocarpus rotundifolius*/*Combretum colinum* woodland. 34. Punda Maria Sandveld on Waterberg sandstone. 35. *Salvadora angustifolia* floodplains.

Our study focused on Kruger National Park, home to approximately 20% and 45% of the world’s black and white rhinos, respectively [4]. The Park is situated in the low-lying savannas of the eastern parts of South Africa and covers an area of 19,485 km^2^. It has annual rainfall of 450–750 mm of which most falls during October to March [10]. Granite and gneiss deposits dominate the west with nutrient-rich basalts in the east, while Karoo sediment characterise where granite and basalt deposits join. These soil types combined with wooded savanna comprising *Sclerocarya caffra* and *Acacia sensu lato nigrescens* on southern basalts, mixed *Combretum spp*. and *Acacia sensu latu spp*. on southern granites and *Colophospermum mopane* in the north define 35 landscapes [11] (Fig 1).

### Poaching and rhino predictions

Detecting carcasses make use of primarily two sources of observations that informed field rangers: field ranger patrols and aerial patrols. From observations, field rangers would visit carcasses and prepare poaching records as part of their field reports. Rangers found carcasses using ground-based patrols of 22 Sections of Kruger National Park. Each section has between 18 and 25 field rangers responsible for patrolling. Rangers made use of local knowledge of the landscape and location of key resources such as seasonal dependent water availability. They also used bush-craft skills such as observing vultures and vulture behavior.

Two helicopter pilots located in Skukuza and two micro-light aircraft pilots located at Tshokwane in southern and Shingwedzi in northern Kruger National Park respectively, complimented field ranger observations. Pilots typically have daily flights over various parts of Kruger. They recorded carcasses opportunistically during flights for a variety of conservation purposes such as aerial surveys or anti-poaching raids. Pilots provided field rangers with information of new carcass discoveries. Field rangers then investigated.

When encountering detected carcasses, field rangers recorded geographical positions, identified the species and assigned an assessment of carcass age based on level of decomposition. Rhino skeletons do not disappear quickly and are detectable even if the carcass is relatively old. We argue that a rhino carcass will eventually be found through various means of observation as described above because rhinos skulls, in particular, has long persistence in the field. Because carcass detection is time dependent, we extracted data for carcasses that had the time since the rhino was killed assigned. We assumed that most carcasses will be detected through the various ways described above within one year of the rhino being killed. This means we could back calculate how many total carcasses were available to be found over a year period since a rhino died. This allowed us to express carcass detection rates as the known number of rhinos found 7 days, 14 days, one month, two months and three months since a rhino died as a fraction of the total carcasses available to be found over a year period since a rhino died.

The last survey for black rhinos using a block-survey design was conducted during 2008 and estimated that 627 (95% CI: 588–666) animals resided in Kruger [5]. We collated the number of black rhino’s poached every year from 2008 to 2012 (SANParks, unpublished data) and converted these to the average number of rhinos poached per day per year. An exponential model [12] fitted to this data predicted the expected daily poaching rate for 2013. Using the observed annual poaching rates, we subtracted these from randomly drawn values extracted from the statistical distributions of the population estimate [5], and allowed the population to increase annually at a rate of 6.75% (95% CI: 4.1–9.8%) [5]. We continued this annual process until we reached a prediction for 2013. By simulating such poaching and population growth effects iteratively (100000 repetitions) [13], we could define the statistical distribution (95% CI) of expected numbers of black rhinos in Kruger during 2013.

During 2012, SANParks conducted aerial distance sampling surveys of herbivores [14] during which observers noted white rhinos as well. The survey had an effective coverage of 6% of Kruger and estimated 10495 white rhino individuals (95% CI: 8500–12900) living in Kruger during 2012 [15]. We allowed annual rates of increases of population to vary between -9.6% and 9.0% predicted from the 2010 and 2012 estimates. We extracted values from the statistical white rhino estimate distribution during 2010 (10,621; 95% CI: 8,767–12,682; [6]), and used daily poaching predictions of white rhino from 2008 to 2012, together with management removal records (SANParks, unpublished data) to derive the expected white rhino population size for 2013. Again, using 100,000 iterative simulations [13], we could define the expected statistical distribution (95% CI) of white rhinos in Kruger during 2013.

Note that annual surveys typically take place during September, the hot dry season. We thus needed to calculate poaching incidence between two surveys. Our results for poaching detection suggest that most carcasses may be detected within three months of the poaching incident taking place (see below). Our collation of poaching data up to the end of 2013, three months after the last population survey, may thus accommodate carcass detection challenges when estimating poaching rates.

### Rhino survey

When estimating abundances of species, several sources of error prevent authorities from obtaining unbiased counts [16, 17]. We used sample-based aerial surveys [18] which carries an inherent source of error [19] because individuals rarely are distributed homogeneously across landscapes [20]. Confidence intervals around estimates [18] capture this error. By increasing the survey effort (percentage of study area randomly covered), confidence intervals generally decrease [21, 22]. Optimal efficiency depends on animal density [23]. A 40% coverage of a focal area will provide most cost-effective confidence intervals for the higher density of white rhinos [6], but may be suboptimal for the lower densities noted for black rhino [5].

At the end of the dry season during 2013, we used block-based surveys [5] by intensely searching 878 blocks 3x3 km in size (41.5% coverage of Kruger) using a Eurocopter Squirrel helicopter. We distributed blocks randomly throughout Kruger National Park, with a slight bias towards the high-density areas south of the Sabie River. Our flights were at an altitude of 45 m above ground level at an average speed of 65 Knots. We divided each block into strips 400 m wide with a 200 m wide search area each side of the aircraft. We minimized double counting by systematically completing transects on a block. Two observers including the pilot on either side of the helicopter with one recorder noted rhinos encountered. Stratifying our samples into landscapes [11] and using Jolly’s estimator [18] allowed landscape-specific estimates and smaller confidence intervals for overall rhino estimates in Kruger.

As the accuracy (a measure of how close an estimate is to the real number of individuals in a population, [24]) of a population estimate comprises of precision (confidence intervals) and bias [25] of the survey methodology, we recognized three biases that could influence the accuracy of estimates. Availability bias [16, 17] estimates the proportion of animals not available to the survey observers, such as those rhinos standing under trees. We checked rhinos that were available, but not detected by sampling 56 white rhinos and 23 black rhinos in localities with varying tree cover [26]. For two black rhino and ten white rhino observations when an individual was already moving, we had the pilot hover and follow the rhino at the same height as our surveys for 10–15 minutes and noted the fraction of time during an observation that the rhino was visible. For cases where rhinos were sedentary (black rhino: *n* = 21; white rhino: *n* = 46) we completed a full circle with radius approximately 100 m at the same height as our surveys and again noted the fraction of time during an observation that the rhino was visible. We then calculated mean percentage time visible for 5% bins of woody cover and fitted an inverse sigmoid curve [27] to these estimates to define the relationship between availability bias and woody cover. We acknowledge, however, that numerous factors may influence the response of a rhino to a helicopter and hence availability bias.

To account for observers having different capabilities, we extracted previously published observer bias estimates [5]. By placing two observers on the same side of an aircraft and allowing independent recording of sightings, we previously [5] used Seber’s approach [28] to estimate that 3.8% of available rhinos to be seen will not be noted by either observer. Finally, we minimized detectability bias (individuals are present and available, but there is variation in detecting them, [14]) by our approach to intensely search blocks using flight paths 400 m apart (*i*.*e*. swath search widths on either side of the aircraft was 200 m). Distance-sampling approaches identified 200 m swath widths as minimally influenced by detectability [29]. Using Monte-Carlo simulations [13], we corrected population estimates for availability and observer bias through 100,000 iterations and define population estimates (median) and confidence intervals (2.5% and 97.5% percentiles) from the resultant statistical distribution.

### White rhino demographics

To ascertain the demographic makeup of the white rhino population in Kruger, we conducted separate aerial based surveys aimed at assigning sex and age to a sample of white rhinos living south of the Olifants River in Kruger. Pilots flew at a height of 45 m at a speed of 60 Knots using a helicopter. Survey teams (pilot and three observers) systematically searched identified areas within individual river catchments. For each rhino group encountered, the pilot reduced height allowing surveyors to obtain better visuals from which they recorded the sex and age of each individual using a body size scale [30, 31], noted geographical position and made a photographic record of each group. GPS recorded flight paths avoided double sampling individuals within an identified area. We collated data from four such demographic surveys since 2009 (October 2009, October 2010, October 2011 and November 2012) as a representative sample of the age- and sex-distribution for each year. For 2013, we used the age- and sex-assignments of the sample of white rhinos encountered on the 878 blocks surveyed then.

We classified individuals into six age-sex classes. This allowed us to expand and smooth sex-specific age-distributions using $\sum_{x}^{w}n_{x}=n_{0}a^{x}\left[\frac{\left(1-a^{\left(w-x+1\right)}\right)}{\left(1-a\right)}\right]$ [32], as the sum of frequencies of individuals that were *x* to *w* years old with *w* the lifespan of white rhinos assumed to be 40 years [33]. Here *n*_*x*_ = number of individuals *x* years old. Frequencies decay at a rate *a*, our smoothing parameter, as age increases (*a* = *s*/*λ*, *s* = survival, *λ* = finite growth rate; [34]). We defined a series of $\sum_{i=x}^{w}n_{i}$ by increasing *x* at increments of 1 up to 7 and setting *w* = 40. We then estimated the decay rate (*a*) across all white rhino ages using maximum likelihood and assuming a normal distribution [35, 36]. Given that we previously predicted no change in population estimates since 2008 [6], we set finite growth rate (*λ*) at one after checking against recent population estimates. Estimated decay rate (*a*) is thus an estimate of survival (*s*).

Removals by managers and poachers [6] may, however, influence standing age distributions. Estimates of mortality (1-*s*_*t*_) in a particular year *t* are thus estimates of total loss rate. We collated annual game capture and poaching data and calculated removal (*r*_*t*_) and poaching (*p*_*t*_) rates as the total number of individuals removed or poached as a percentage of the estimated number of individuals in the population for each sex respectively. Subtracting these rates from the total loss rate provided estimates of natural mortality [*m*_*t*_ = (1-*s*_*t*_)-*r*_*t*_-*p*_*t*_]. Recruitment (*b*_*t*_) was the number of individuals younger than one year as a percentage of the total number of individuals noted for a specific sex.

We used least square linear regression analyses [12] to check how rates changed over time. We were also interested in how rates may vary with rainfall and population size. For rainfall indices, we extracted monthly records from three weather stations (Kingfisherspruit, Stolsnek and Crocodile Bridge taken to be representative of the southern region of Kruger) and estimated the average rainfall in the preceding year. For population sizes, we had to derive estimates for 2009 and 2011, years during which no formal estimates were available. Here we used the average point estimates of the year preceding and year after each missing estimate. Although this introduced non-independence, we used linear regression analyses [12] to check how estimated mortality and recruitment rates associated with rainfall and population size. Model selection criteria [37] helped to identify the most likely fitting model.

The final demographic analyses sought to elucidate the demographic effect of poaching as indicated by relationships between population growth rates and population sizes. We calculated predicted population growth for the present status quo (*b*_*t*_−*s*_*t*_); conditions with poaching, but no management [*b*_*t*_−*m*_*t*_−*p*_*t*_]; conditions with management, but no poaching [*b*_*t*_−*m*_*t*_−*r*_*t*_]; and conditions with no management and no poaching [*b*_*t*_−*m*_*t*_] from the estimated mortality (*m*_*t*_), recruitment (*b*_*t*_), removal (*r*_*t*_) and poaching (*p*_*t*_) rates annually. We then related these various estimated population growth rates to population sizes using linear regression analyses [12].

### Predicting future trends

We used the estimated population size as well as noted sex ratios of white rhinos recorded during 2013 to make predictions over a 5-year horizon. Our interest is primarily short- to medium term, a time frame that typically overlaps with conservation management plans. We define recruitment rates as well as natural mortality rates, each with statistical distributions using the vital rates extracted from demographic observations. We allowed the sex-specific populations at time *t* to recruit new rhinos by drawing rates randomly from the defined recruitment distributions. Similarly we allowed a proportion of rhinos alive at time *t* to die between time *t* and *t*+1 based on the distribution of natural mortality rates that we estimated from age distributions. We then subtracted the number of rhinos expected to be poached between time *t* and *t*+1 to obtain population estimates of males and females and ultimately the total population. We repeated this process starting in 2013 and ending during 2018. Using 100,000 iterative simulations [13], we could define the expected statistical distribution (95% CI) of white rhino population estimates in Kruger during 2018.

We considered four scenarios for expected poaching. For the first scenario we anticipated that poaching trends noted from 1998 to 2013 will continue. Expected poaching rates thus came from the observed rate of poaching (rhinos poached as a fraction of the population sizes) recorded from 1998 to 2013. An exponential model [12] fitted to this data predicted the number of rhinos poached for a specific year. For the second scenario we anticipated that the exponential rate of increase in poaching is half of what the case was by the end of 2013. Scenario three assumed that the poaching rate observed during 2013 stays the same for the next 5 years, while scenario four assumed poaching rate over the next five years is half of what it was during 2013.

## Results

### Predicted results

**Fig 2:**
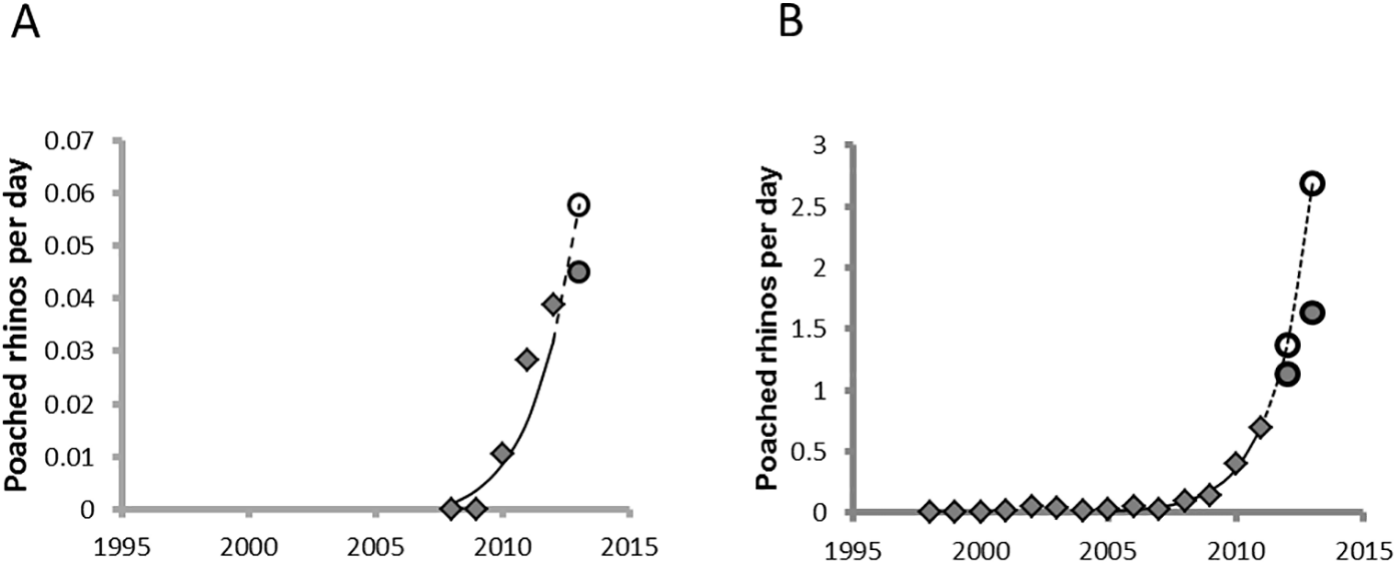
Rhino poaching rates in Kruger National Park. Observed and expected daily poaching rates for black (A) and white (B) rhinos in Kruger National Park. The diamonds are previously reported poaching rates. The open circles the predicted rates derived from the historic trends with the filled circles the observed rates noted during the present study.

Poaching rates (number of rhinos killed per day) increased exponentially on an annual basis since the mid-2000s. For black rhino, our model (*y* = 0.002*e* ^0.633*x*^ *e* ^−0.074*x*^, *r* ^*2*^ = 0.96) predicted a black rhino poaching rate of 0.06 rhinos per day during 2013 (or 22 for the year). Using previous models [6], the poaching pressure for white rhinos was predicted to be significantly higher with 2.69 rhinos expected to be killed per day during 2013 (or 982 for the year) (Fig 2).

Predicted black rhino population estimates given observed and predicted poaching since 2008 was 835 (95% CI: 754–956). In contrast, given observed and predicted poaching and population estimates during 2010, we predicted 9,417 (95% CI: 7,698–11,183) white rhinos should be living in Kruger during 2013 if poaching, management and population dynamics followed the same trends noted up to 2010.

### Observed results

Carcass detection improved as time progress since a poaching event has taken place. From 2010 to 2013 SANParks rangers detected on average 63% of carcasses within 7 days since a poaching event took place. Three months after a poaching event, rangers have detected between 94% and 99% of carcasses since a poaching event took place (Table 1).

**Table 1:** Carcass detection rates estimated for 2010, 2011, 2012 and 2013 using assigned ages of carcasses (*i*.*e*. time since poaching event took place).

	Carcass Detection Rates				
Time since poaching event	2010	2011	2012	2013	Average
	(*n* = 103)	(*n* = 167)	(*n* = 326)	(*n* = 266)	(95% CI)
1 week	0.67	0.73	0.56	0.55	0.63
					(0.54–0.71)
2 weeks	0.82	0.84	0.73	0.73	0.78
					(0.72–0.83)
1 month	0.87	0.93	0.85	0.91	0.89
					(0.86–0.93)
2 months	0.95	0.98	0.90	0.97	0.95
					(0.92–0.98)
3 months	0.98	0.98	0.92	0.97	0.96
					(0.94–0.99)

We also provide the average detection rates across all four years and the number of carcasses for which time since death estimates were available.

**Fig 3:**
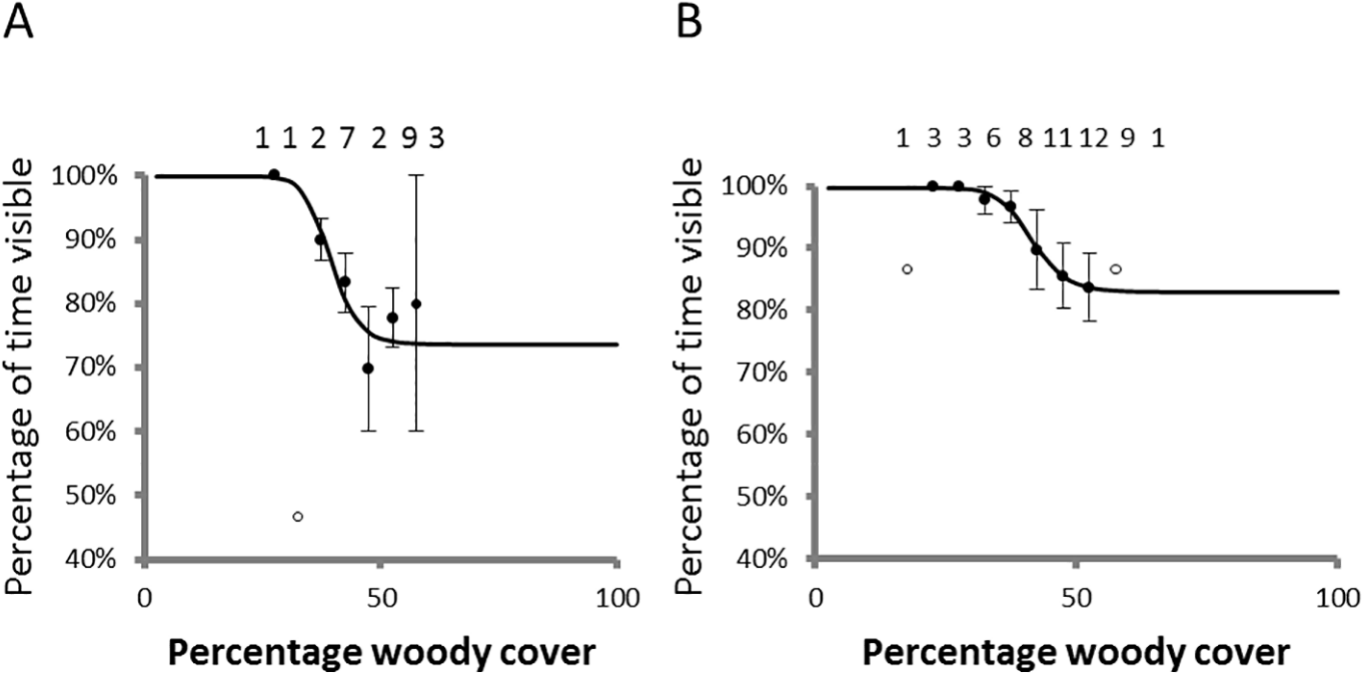
Availability bias for rhinos in Kruger Natonal Park. Availability biases for black (A) and white rhinos (B) are expressed as the percentage time visible against woody cover of a locality. Open symbols represent outliers typically when only one sample was available. Lines represent fitted inverse sigmoid curves.

Observed poaching rates were lower than that predicted from previous years trends for both species (Fig 2). Surveyors actually encountered 3,873 white rhino and 169 black rhino on the 878 surveyed blocks in Kruger. These observations were constrained by availability bias. Our models predicted that as low as 73.6% and 82.9% of black and white rhinos respectively, were available to be sampled depending on woody cover of a sample block (black rhino: $y=1-\left[0.001-\frac{0.264-0.001}{1+{\left(\frac{x}{34.41}\right)}^{-13.63}}\right]$, *r* ^*2*^ = 0.841; white rhino: $y=1-\left[0.003-\frac{0.171-0.003}{1+{\left(\frac{x}{41.47}\right)}^{-12.78}}\right]$, *r* ^*2*^ = 0.987, where *x* is woody cover and *y* the percentage of time a rhino is visible, Fig 3).

**Fig 4:**
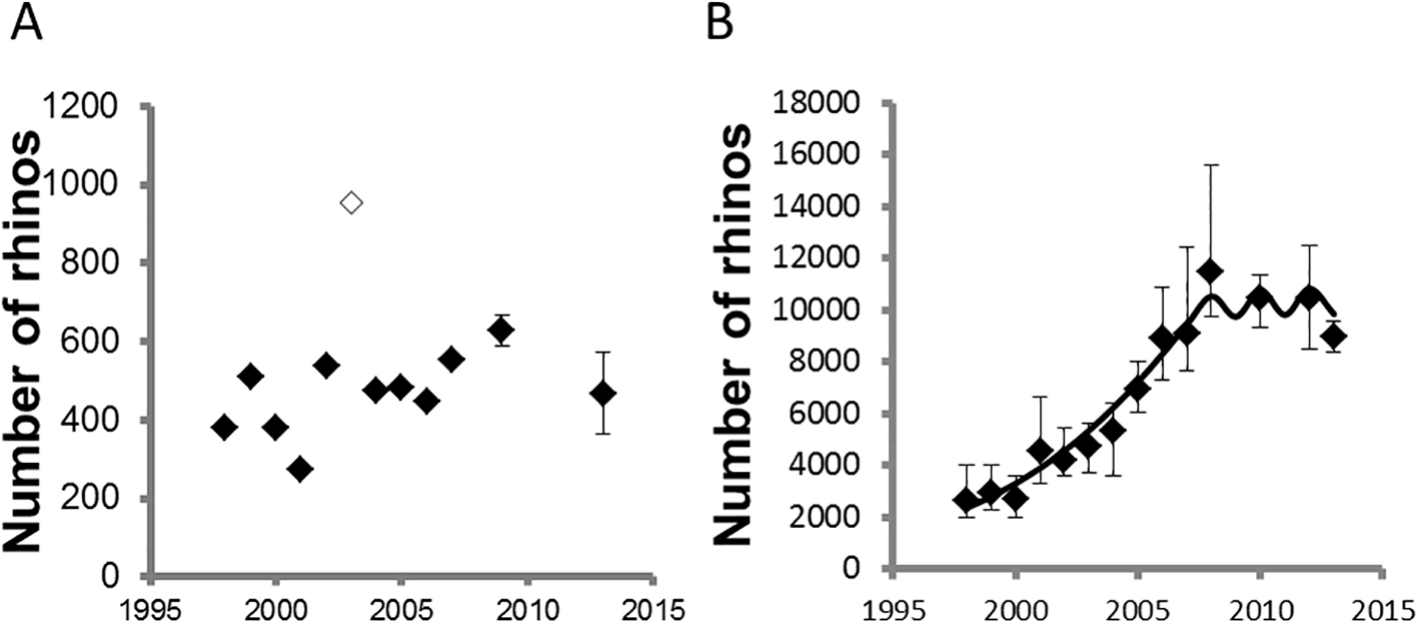
Population trends for rhinos in Kruger National Park. Black (A) and white (B) rhino estimates noted in Kruger National Park. We extracted previous estimates for black rhino [5] and white rhino [9, 15]. The line for white rhino comes from a published model [9].

Landscape-specific black rhino estimates (12 landscapes had blocks with black rhino present) inclusive of biases varied considerably with the highest abundance (141, 95% CI: 101–181) and density (0.11, 95% CI: 0.08–0.15 *n*.*km* ^-2^) in thickets of the Sabie and Crocodile River (Table 2). White rhinos were more widespread with 24 landscapes having at least one or more blocks noted with white rhinos present. Landscape-specific white rhino estimates noted highest abundance in mixed *Combretum* / *Terminalia sericea* woodland (1,921, 95% CI: 1,645–2,234), but highest density in *Combretum collinum* / *Combretum zeyheri* woodland (2.19, 95% CI: 1.79–2.61 *n*.*km* ^-2^) (Table 2). Overall estimates for black rhino (414, 95% CI: 343–487) was considerably lower than that predicted. We noted, however, that the 2013 black rhino estimate had overlapping confidence intervals with eight of the eleven estimates since 1998. White rhino estimates (8968, 95% CI: 8394–9564), in contrast, had overlapping statistical distributions with those predicted. We noted that the 2013 white rhino estimate had overlapping confidence intervals with those since 2006 (Fig 4) implying that white rhino number had little detectable change over that period.

**Table 2:** Landscape-specific estimates and densities of black and white rhino recorded in Kruger National Park during 2013.

	White Rhino		Black rhino	
Landscape	Estimate	Density	Estimate	Density
01: Lowveld Sour Bushveld of Pretoriuskop	477	1.28	24	0.06
	(358–598)	(0.96–1.61)	(1–47)	(0.00–0.13)
02: Malelane Mountain Bushveld	776	1.64	51	0.11
	(575–987)	(1.22–2.09)	(26–79)	(0.06–0.17)
03: *Combretum collinum* / *Combretum zeyheri* Woodland	987	2.19	28	0.06
	(804–1174)	(1.79–2.61)	(12–44)	(0.03–0.10)
*04: Thickets of the Sabie & Crocodile River	1585	1.28	141	0.11
	(1338–1839)	(1.08–1.48)	(101–181)	(0.08–0.15)
05: Mixed *Combretum* / *Terminalia sericea* Woodland	1921	1.29	52	0.04
	(1645–2234)	(1.11–1.51)	(23–83)	(0.02–0.06)
06: *Combretum* / *Colophospermum mopane* Woodland of Timbavati	222	0.55	0	0
	(130–317)	(0.33–0.79)	-	-
07: Olifants River Rugged Veld	70	0.14	0	0
	(32–107)	(0.06–0.21)	-	-
08: Phalaborwa Sandveld	24	0.06	0	0
	(0–60)	(0.00–0.15)	-	-
09: *Colophospermum mopane* Woodland / Savanna on Basic Soil	58	0.14	0	0
	(32–84)	(0.08–0.21)	-	-
10: Letaba River Rugged Veld	0	0	0	0
	-	-	-	-
11: Tsende Sandveld	51	0.03	0	0
	(21–81)	(0.01–0.05)	-	-
12: *Colophospermum mopane* / *Acacia nigrescens* Savanna	126	0.12	0	0
	(60–193)	(0.06–0.19)	-	-
13: *Acacia welwitschii* Thickets on Karoo Sediments	219	0.45	30	0.06
	(143–301)	(0.30–0.62)	(13–49)	(0.03–0.10)
14: Kumana Sandveld	125	1.08	9	0.08
	(85–165)	(0.73–1.43)	(2–16)	(0.02–0.14)
15: *Colophospermum mopane* Forest	0	0	0	0
	-	-	-	-
16: Punda Maria Sandveld on Cave Sandstone	11	0.06	0	0
	(0–27)	(0.00–0.14)	-	-
17: *Sclerocarya birrea* subspecies *caffra* /*Acacia nigrescens* Savanna	798	0.59	18	0.01
	(649–952)	(0.48–0.70)	(3–32)	(0.00–0.02)
18: Dwarf *Acacia nigrescens* Savanna	188	0.55	3	0.01
	(123–254)	(0.36–0.54)	(0–7)	(0.00–0.02)
19: Thornveld on Gabbro	735	1.01	30	0.04
	(558–928)	(0.76–1.27)	(10–51)	(0.01–0.07)
20: Bangu Rugged Veld	75	0.36	0	0
	(55–96)	(0.26–0.46)	-	-
21: *Combretum* / *Acacia nigrescens* Rugged Veld	30	0.13	7	0.03
	(5–55)	(0.02–0.23)	(0–15)	(0.00–0.06)
22: *Combretum* / *Colophospermum mopane* Rugged Veld	12	0.02	0	0
	(0–28)	(0.00–0.03)	-	-
23: *Colophospermum mopane* Shrubveld on Basalt	47	0.02	0	0
	(24–71)	(0.01–0.04)	-	-
24: *Colophospermum mopane* Shrubveld on Gabbro	38	0.12	0	0
	(0–82)	(0.00–0.27)	-	-
25: *Adansonia digitata* / *Colophospermum mopane* Rugged Veld	0	0	0	0
	-	-	-	-
26: *Colophospermum mopane* Shrubveld on Calcrete	0	0	0	0
	-	-	-	-
27: Mixed *Combretum* / *Colophospermum mopane* Woodland	0	0	0	0
	-	-	-	-
28: Limpopo / Luvuvhu Floodplains	0	0	0	0
	-	-	-	-
29: Lebombo South	362	0.46	21	0.03
	(263–470)	(0.34–0.60)	(8–35)	(0.01–0.04)
30: Pumbe Sandveld	-	-	-	-
	-	-	-	-
31: Lebombo North	30	0.05	0	0
	(0–77)	(0.00–0.14)	-	-
32: Nwambiya Sandveld	0	0	0	0
	-	-	-	-
33: *Pterocarpus rotundifolius* / *Combretum collinum* Woodland	0	0	0	0
	-	-	-	-
34: Punda Maria Sandveld on Waterberg Sandstone	0	0	0	0
	-	-	-	-
35: *Salvadora angustifolia* Floodplains	0	0	0	0
	-	-	-	-

Kruger National Park had a total of 8968 (95% CI: 8394–9564) white rhinos and 414 (95% CI: 343–487) black rhinos during 2013. The values in brackets are 95% confidence intervals.

### White rhino demography

**Fig 5:**
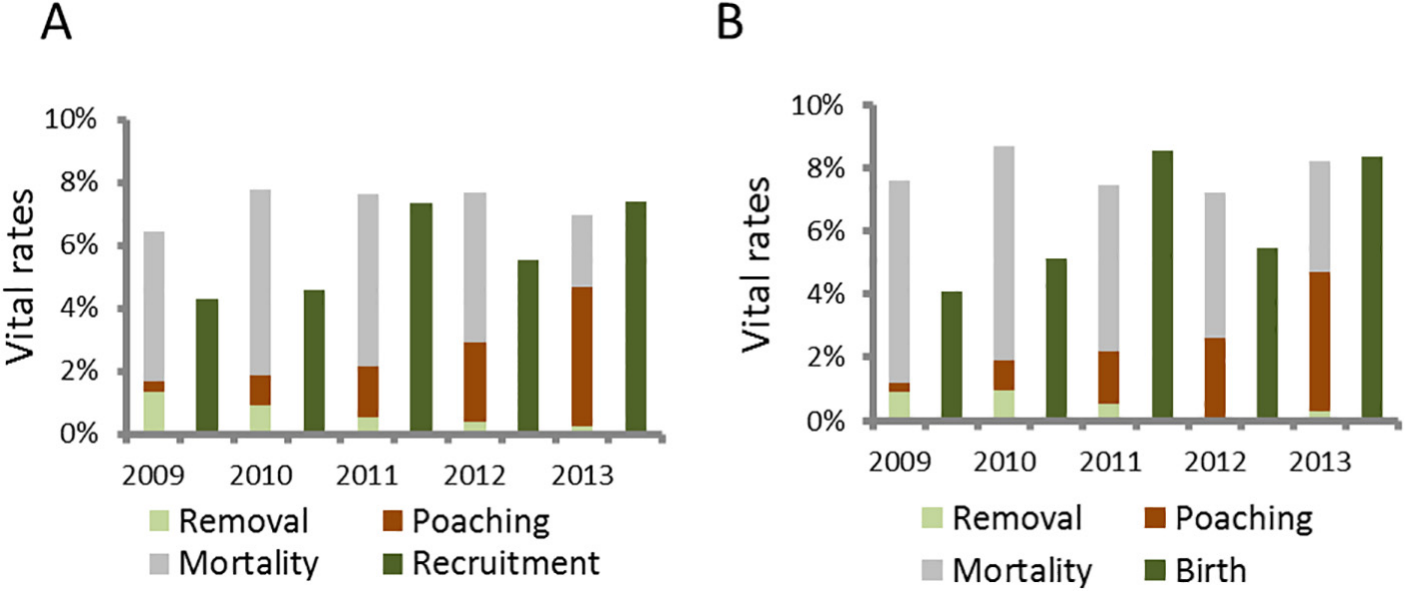
Estimated vital rates for white rhinos noted in Kruger National Park. We present data for males (A) and females (B) since 2009. We also include management removal and poaching rates.

We sampled 1477 (2009), 1512 (2010), 1426 (2011), 996 (2012) and 3873 (2013) white rhinos for which we assigned ages and sexes. Management removal rates declined from 2009 onwards (♂♂: *F*_*1*,*3*_ = 11.61, *P* = 0.04; ♀♀: *F*_*1*,*3*_ = 34.69, *P* = 0.01), while poaching rates increased (♂♂: *F*_*1*,*3*_ = 45.75, *P* < 0.01; ♀♀: *F*_*1*,*3*_ = 44.88, *P* < 0.01) (Fig 5). Although natural mortality rates of males declined since 2009 (♂♂: *F*_*1*,*3*_ = 25.72, *P* = 0.01), non-directional variation in female mortality (♀♀: *F*_*1*,*3*_ = 2.66, *P* = 0.20) and rhino recruitment rates (♂♂: *F*_*1*,*3*_ = 2.85, *P* = 0.19; ♀♀: *F*_*1*,*3*_ = 4.28, *P* = 0.13) resulted in average annual loss rates (♂♂: 7.8%, 95% CI 7.3%–8.4%; ♀♀: 7.3%, 95% CI 6.8%–7.8%). These had overlapping confidence intervals compared to average annual recruitment rates (♂♂: 6.3%, 95% CI 4.5%–7.8%; ♀♀: 5.8%, 95% CI 4.5%–7.1%) for both males (*t*_*8*_ = 1.60, *P* = 0.15) and females (*t*_*8*_ = 2.09, *P* = 0.08) since 2009.

**Fig 6:**
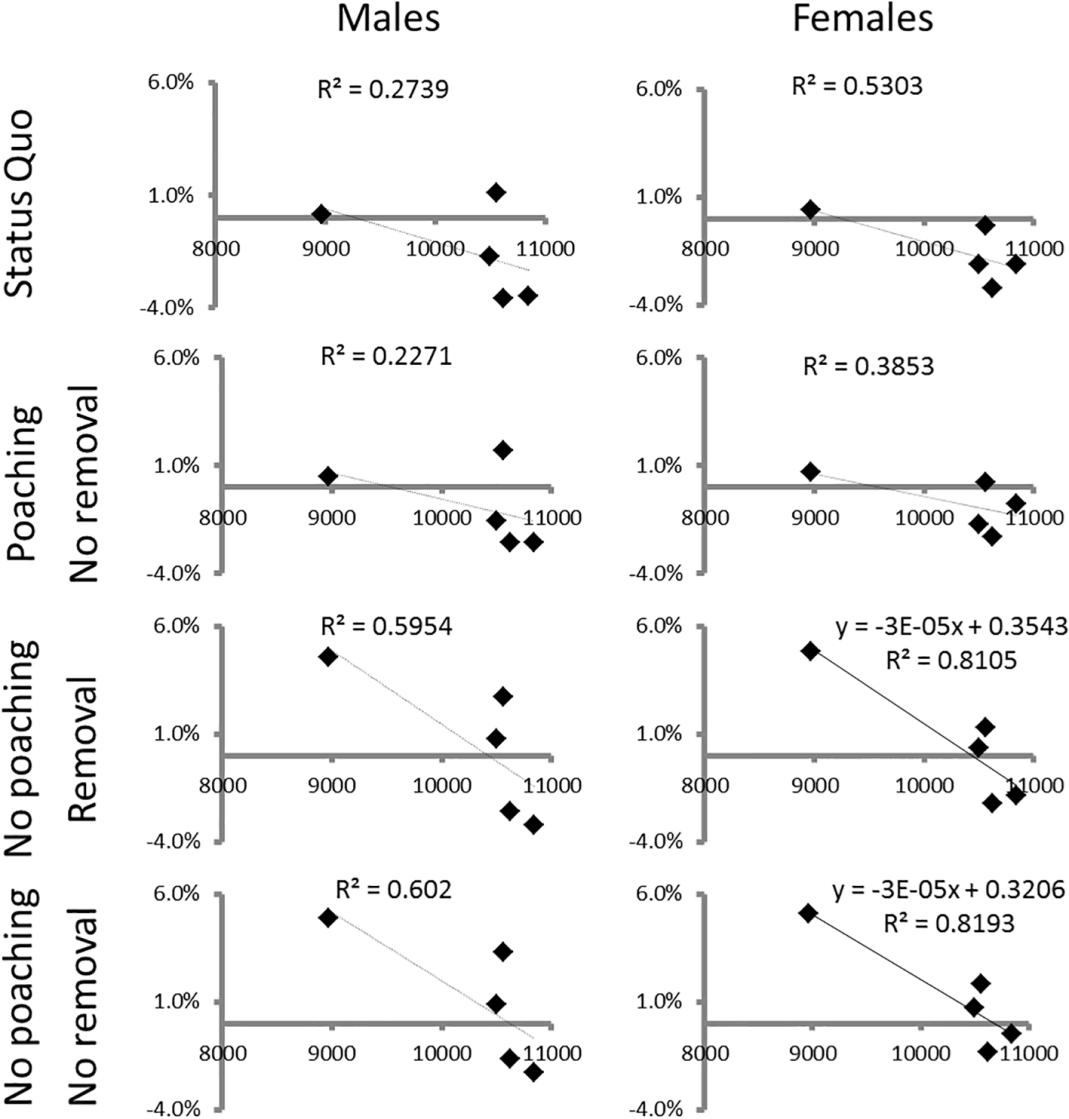
Estimated annual population growth rates as a function of population size. We illustrate how poaching and management removals may influence these relationships. Solid lines are significant relationships.

Female mortality rates increased with population size while male mortality decreased with rainfall. Although small samples influenced reliable model selection, those that included rainfall and population size explained recruitment rates best, irrespective of sex (Table 3). Predicted annual growth rates from balances between loss and recruitment rates highlighted disruption of demographic population responses by poaching (Fig 6). In the absence of poaching, demographic responses predicted strong density-dependence of population growth particularly for females.

**Table 3:** Explanatory capabilities of previous year rainfall and population size for variance in births and deaths as indicated by model selection procedures.

Males	Births				Deaths			
Candidate model	*AIC*_*i*_	*∆*_*i*_	*L*_*i*_	*w*_*i*_	*AIC*_*i*_	*∆*_*i*_	*L*_*i*_	*w*_*i*_
Rainfall + Population size	**-45.07**	**-**	**1.00**	**0.76**	-46.10	1.95	0.38	0.19
Rainfall	-42.36	2.71	0.26	0.20	**-48.05**	**-**	**1.00**	**0.51**
Population size	-39.43	5.63	0.06	0.05	-46.93	1.12	0.57	0.29
Females	Births				Deaths			
Candidate model	*AIC*_*i*_	*∆*_*i*_	*L*_*i*_	*w*_*i*_	*AIC*_*i*_	*∆*_*i*_	*L*_*i*_	*w*_*i*_
Rainfall + Population size	**-48.91**	**-**	**1.00**	**0.90**	-44.90	1.93	0.38	0.22
Rainfall	-44.22	4.69	0.10	0.09	-44.88	1.95	0.38	0.21
Population size	-40.62	8.27	0.02	0.01	**-46.84**	**-**	**1.00**	**0.57**

*AIC*_*i*_ is the Akaike Information Criterion value for candidate model *i*. For each variable, we considered three candidate models that included combinations of rainfall and population size. *∆*_*i*_ represent the difference in *AIC*_*i*_ with the minimum *AIC*_*i*_ noted for candidate models. Models have a high likelihood (*L*_*i*_) and weight of evidence (*w*_*i*_) when *∆*_*i*_>2. We highlight in bold candidate models with greatest weight of evidence.

### Future trends

If poaching trends noted by 2013 continues, the white rhino population in Kruger will decline to between 2879 and 3263 individuals by 2018, the worst case scenario that we considered (Table 4). Halving the poaching rates of 2013 will result in 7608 to 8759 white rhinos in Kruger by 2018, similar to the population size estimated for 2013.

**Table 4:** Predicted white rhino population estimates for 2018 under four different scenarios for poaching trends.

	Estimate	LCL	UCL
Present rise in poaching rates continues	3076	2879	3263
Present rise in poaching rates halved	6691	6172	7271
Present rate of poaching sustained	6886	6344	7476
Present rate of poaching halved	8106	7608	8759

LCL–Lower 95% Confidence Limit. UCL–Upper 95% Confidence Limit.

## Discussion

The poaching onslaught on Africa’s rhinos [3] has not abated–at least not in Kruger National Park, a globally important stronghold for both the white and the black rhino [4]. The white rhino estimates we provide in this study suggest that the high annual growth rates observed between 1998 and 2008 (Fig 4) and for many years before that [6], were not sustained in recent years. In fact, population estimates since 2008 suggest that the recent annual growth rates for white rhino is fluctuating around zero, with the latest estimate in 2013 suggesting that the growth rate may even be negative or at least fast approaching negative values. We attribute much of this rapid drop in growth rate to substantial increase in illegal poaching since 2009 (Fig 2 & Fig 5). As a result of the escalation in poaching, SANParks purposefully reduced management removals (mostly with the objectives of species range expansion and generating revenue for conservation [4, 6]) from 246 white rhinos in 2009 to 50 in 2013 (Fig 5). Without this reduction in management removals in recent years, our results predict significant negative growth rates in white rhino numbers in Kruger by 2013. If the poaching onslaught does not decrease and the poaching rates continue to rise at current levels, we estimate that white rhino population will plummet to 2879 to 3263 individuals (95% CI) by 2018, about a third of the 2013 estimate of 8394 to 9564 individuals (95% CI) (Table 4). If authorities can contain or reduce poaching rates the situation is somewhat better, but still worrisome as the population will still decrease from current estimates (Table 4).

The trend for black rhino population estimates is less clear. Black rhino is a low density species in Kruger [5] and often prefer denser habitats (*i*.*e*. highest densities observed in the dense Sabie/Crocodile thickets–Table 2). Furthermore, the visibility and thus availability of black rhino for observing during surveys is lower, be it due to habitat or behaviour, and more variable than for the white rhino (Fig 3). These characteristics make it challenging to estimate black rhino population numbers reliably in Kruger from aerial surveys. Considering that most past surveying efforts for black rhino did not provide confidence levels for the population estimates [5], combined with the fact that sampling effort was not adequate [23] to estimate such a low density species with variable availability, caution should be exercised when interpreting black rhino trends. Therefore, based on the best available black rhino estimates from recent years, there is no clear indication of a population trend in Kruger (1997–2013).

The reduced black rhino estimate of 2013 compared to 2009 [5]—covering the period when rhino poaching intensified in Kruger (Fig 2)—does flag some concern. As such, authorities need estimates based on consistent and improved sampling techniques to define black rhino population trends. It will remain highly unlikely to get cost-effective and reliable population estimates for Kruger’s black rhino using standard aerial survey techniques [17, 18, 20]. The use of focal individuals to derive estimates of vital rates [38, 39, 40] may be a useful method with which to supplement population estimates. Although individual recognition through dedicated observation, like the registration studies employed in smaller reserves [38, 39, 40], is not feasible in areas the size of Kruger, registration studies in black rhino hotspots within Kruger may complement aerial survey approaches by using tracking devices fitted to a sample of black rhino individuals [41] to facilitate evaluation of vital rates.

It is not only management removals or poaching that influence rhino demographics. Theoretical models [42] predict increased juvenile mortality, followed by fecundity reduction and ultimately increased adult mortality when large herbivores experience density-dependent regulation. As such, we anticipate that, for white rhinos, birth rates may decline with increased density and decreased rainfall as predicted by these models. Our data supported some of these predictions albeit with some uncertainty. We also suspect, however, that increased poaching [3] will most likely disrupt such demographic responses—*i*.*e*. poachers target adults rather than juveniles [6]. It is arguable whether these density-dependent processes described above are acting in Kruger as the white rhino densities are still relatively low compared to other systems like. For instance, at Hluhluwe-iMfolozi Park, where white rhinos live at higher densities than at Kruger [43], there is no evidence for density-dependent responses in vital rates [44]. Nonetheless, our results provide some indication that rainfall and density influence vital rates for Kruger’s white rhinos, with growth rates positively related to rainfall and negatively with population density (Table 3 & Fig 6).

The responses of white rhinos implied by our results highlight various ways in which authorities can use strategic removals and translocation of rhinos, in space and time, to increase birth rates and/or decrease mortality rates. Firstly, strategic removal of rhinos from focal areas that are heavily targeted by poachers, due to the difficulty in protecting them in those specific locations, to areas of lower risk, could reduce mortality rates. For example, it may be advisable to move rhinos from poaching hotspots close to international boundaries that provide ample escape opportunities for the poachers towards areas of lower risk further from the park boundaries and with easier access for management patrols and anti-poaching operations. Such removals also have the additional benefit of focusing management actions over smaller geographical areas.

Secondly, strategic rhino removals from focal landscapes with relatively high densities where environmental and density-dependent population regulation may be operating, can possibly contribute to stimulating population growth rates in those landscapes through increased birth rates and decreased mortality rates. This is in line with the constant harvest strategy advocated for high density black rhino populations [45]. In such a case, authorities translocate rhinos from such high density areas into intensely protected sanctuaries and areas owned by private individuals or local community areas [7]. Such translocations could offset anticipated poaching effects in the short- to medium term through induced lower mortalities and higher birth rates because of lower local densities [46]. Additionally, new nuclear populations, possibly with higher recruitment due to intensive protection and management, can be established, as well as widening the ownership basis and hence shared interest in protection of the species. Such strategies may thus result in positive growth rates both in the source and in the recipient sites.

Thirdly, removal and translocation strategies may also want to consider rainfall in the preceding year to adjust annual rhino removal targets as vital rates vary between years and birth rates may be higher and death rates lower in years with more abundant rainfall (with rainfall acting as proxy for available forage and water resources). Removing rhinos in prolonged dry periods from poor habitats to areas of good habitat and higher rainfall may also possibly increase birth rates and decrease mortality rates. Note, that the above strategies are largely based on assumptions currently untested, and need further thorough research within the Kruger socio-ecological context. Even so, biological management responses that facilitated range expansions played a key role in recovering both black [45] and white rhinos [4].

An alternative approach to stimulate population growth rate is to improve habitats for rhino through, for instance timing and location of management fires or density and spatial arrangement of artificial water sources [47]. However, at present it is not clear if, and how, authorities can manage Kruger’s habitats improve rhino vital rates.

Authorities should, however, be cautious when considering the application of “rhino growth rate optimization” actions, as outlined above, for the sake of the individual species. SANParks adheres to a systems-based [48] rather than a species- or issue-based management approach [47]. Rhino conservation actions should therefore not be in conflict with broader systems objectives. Also, management should always be cognisant of the fact that their actions directed at rhinos may have cascading effects beyond the target species. For example, removal of white rhinos in Hluhluwe-iMfolozi Park influenced ecosystem patterns such as the formation and maintenance of grazing lawns as well as landscape processes such as fire spread [43]. An additional risk of a species-based management focus, which is easy to develop when a charismatic species like the rhino is under threat, is that it compromise and detract resources from other priority conservation actions like fire management, vegetation monitoring, benefit sharing and constituency building with neigbouring communities and addressing larger systemic threats (*e*.*g*. acute pollution of freshwater ecosystems). As managers are forced to spend more and more resources on protection of a single species, other conservation issues will receive less attention. Additionally, institutional ability to do certain other conservation management actions that were built over many years of experience starts fading and management can become increasingly one-dimensional.

Biological management such as habitat improvement through directed management actions, and/or assisted colonization, and/or establishment of intensely protected rhino sanctuaries using white rhinos sourced from high-density landscapes or poaching hotspots are heavily dependent on successful protection of white rhinos [2]. Trends in rhinos poached per day noted in the present study suggest that intensive militarization [49] has at best slowed the escalation of poaching rates—although the year-to-year poaching incidences are still on the increase (Figs 2 & 5). While rhino population responses that we noted provide some avenues for exploring the biological management of them, managing the threat posed by poachers is a key complimentary requirement [50] needed to achieve global rhino conservation objectives, which largely focus on increasing population sizes [4].

Integrated approaches [51] to reduce the threat posed by poachers include, over and above the usual military anti-poaching approach, actions such as disruption of international criminal networks [52], attuned legal and extradite agreements between countries targeted by poachers and harbouring poachers and horn dealers [51], reduction in illegal demand for horn [51], and provision of alternative economies in communities where poaching originates [53]. It is clear that the current strategy of militarized anti-poaching effort [49], although needed and most likely slowing the wave of poaching, will not solely address the problem, and also poses risks of its own such as alienating conservation agencies from its neighbouring communities [54]. Rather, a multi-pronged approach [51] is needed, where many options outside the traditional sphere of field-based management and conservation inside park boundaries need to be explored.

## Conclusions

Our results illustrated variable responses of black and white rhinos to variable ecological constraints as well as anthropogenic influences induced by managers and poachers. Sampling effects of a small population may confound our results for black rhinos. Nonetheless, black rhinos are most likely declining. The white rhino population in Kruger may decline in future if poaching trends noted up to 2013 continue. We can only speculate what the situation might have been if no anti-poaching activities took place. The reality is that existing concerted and outstanding anti-poaching efforts [4] has not resulted in reversing the predictions that of future declines of white rhino populations in Kruger [6]. Loss rates and birth rates were largely equal since 2008 primarily because conservation managers could compensate for increasing poaching rates by reducing management removals. High rainfall since 2008 may also have led to increased birth rates and reduced death rates in recent years. Continued poaching rate increases have now removed the “subsidy” afforded by reduced management removals and higher rainfall. We have argued that short- to medium biological management responses have the potential to stimulate population growth rates, but these need to be in concert with sustained present anti-poaching activities. Authorities need to complement both of these with focused intelligence-based disruption of crime networks at various scales. This is particularly important given that crime network disruption is a requirement irrespective of the strategic focus that conservationists may adhere too. The harsh reality is that within Kruger National Park, the populations of both black and white rhino species are most likely declining and will continue to do so unless conservation action addresses a variety of ultimate and proximate drivers of poaching.

## References

[1] Venter FJ, Naiman RJ, Biggs HC, Pienaar DJ (2008) The evolution of conservation management philosophy: Science, environmental change and social adjustments in Kruger National Park. Ecosystems 11: 173–192.

[2] Emslie RH, Brooks M (1999) African rhino: Status survey and conservation action plan Gland: IUCN/SSC African Rhino Specialist Group.

[3] Thomas R (2010) Surge in rhinoceros poaching in South Africa. TRAFFIC Bulletin 23: 3.

[4] Knight MH (2013) African Rhino Specialist Group report. Pachyderm 53: 7–24. 24303297

[5] Ferreira SM, Greaver CC, Knight MH (2011) Detecting population performance in the black rhino population of Kruger National Park, South Africa. S Afr J Wildl Res 41: 192–204.

[6] Ferreira SM, Botha JM, Emmett M (2012) Anthropogenic influences on conservation values of white rhinos. PLOS ONE 7: e45989 10.1371/journal.pone.0045989 23029354 PMC3459945

[7] Carruthers J (2008) Wilding the farm or farming the wild: The evolution of scientific game ranching in South Africa from the 1960s to the present. Trans R Soc S Afr 63:160–181.

[8] Ehrenfeld JG (2000) Defining the limits of restoration: The need for realistic goals. Restor Ecol 8: 2–9.

[9] Pienaar DJ (1993) The landscape preference and horn attributes of the white rhinoceros *Ceratotherium simum simum* (Burchell, 1817) in the Kruger National Park. University of Pretoria: MSc-Thesis.

[10] Venter FJ, Scholes RJ, Eckhardt HC (2003) The abiotic template and its associated vegetation pattern In: du Toit J, Biggs HC, Rogers K. editors. The Kruger experience: ecology and management of savanna heterogeneity. Washington: Island Press pp. 83–129.

[11] Gertenbach WPD (1983) Landscapes of the Kruger National Park. Koedoe 26: 9–121.

[12] Sokal RR, Rohlf FJ (1969) Biometry: The principles and practice of statistics in biological research San Francisco: W.H. Freeman and Company.

[13] Fishman GS (1995) Monte Carlo: Concepts, algorithms and applications New York: Springer.

[14] Buckland ST, Anderson DR, Burnham KP, Laake JL (1993) Introduction to distance sampling: Estimating abundance of biological populations Oxford: Oxford University Press.

[15] Ferreira SM (2013) An update on rhino research and surveys in SANParks Skukuza: SANParks.

[16] Caughley G (1974) Bias in aerial survey. J Wildl Manage 38: 921–933.

[17] Redfern JV, Viljoen PC, Kruger JM, Getz WM (2002) Biases in estimating population size from an aerial census: a case study in the Kruger National Park, South Africa. S Afr J Sci 98: 455–461.

[18] Jolly GM (1969) Sampling methods for aerial censuses of wildlife populations. East Afr Agric For J 34: 46–49.

[19] Walsh PD, White LJT, Mbina C, Idiata D, Mihindou Y, Maisels F, et al (2001) Estimates of forest elephant abundance: projecting the relationship between precision and effort. J Appl Ecol 38: 217–228.

[20] Caughley G (1977) Sampling in aerial survey. J Wildl Manage 41: 605–615.

[21] Barnes RFW (2002) The problem of precision and trend detection posed by small elephant populations in West Africa. Afr J Ecol 40: 179–185.

[22] Grimsdell JJR, Bille JC, Milligan K (1981) Alternative methods of aerial livestock census. In: Grimsdell JJR, Westley SB. editors. Low-level aerial survey techniques. Addis Ababa: ILCA/ILRI Monograph 4.

[23] Ferreira SM, van Aarde RJ (2009) Aerial survey intensity as a determinant of estimates of African elephant population sizes and trends. S Afr J Wildl Res 39: 181–191.

[24] Everitt BS (2002) Cambridge dictionary of statistics New York: Cambridge University Press.

[25] Thompson SK (1992) Sampling New York: John Wiley & Sons.

[26] Bucini G, Hanan NP, Boone R, Saatchi S, Smit I, Lefsky MA, et al (2011) Woody cover in Kruger National Park, South Africa: remote sensing based maps and ecological insights In: Hill MJ, Hanan NP. Editors. Ecosystem function in savannas: Measurement and modeling at landscape to global scales. Boca Raton: CRC Press/Taylor & Francis pp. 218–238.

[27] Han J, Morag C (1995) The influence of sigmoid function parameters on the speed of backpropagation learning. Proceedings Series: Lecture Notes in Computer Science 930: 195–201.

[28] Seber GAF (1982) The estimation of animal abundance Caldwell: Backburn Press.

[29] Kruger JM, Reilly BK, Whyte IJ (2008) Application of distance sampling to estimate population densities of large herbivores in Kruger National Park. Wildl Res 35: 371 *–* 376. 10.1016/j.jchemneu.2008.02.005 18407460

[30] Hillman-Smith K, Owen-Smith RN, Anderson IL, Hall-Martin AJ, Selaladi JP (1986) Age estimation of the white rhinoceros (*Ceratotherium simum*). J Zool 210: 355–379.

[31] Emslie RH, Adcock K, Hansen HB (1995) Fine tuning the rhino management group age class system Rustenburg: Rhino Management Group.

[32] Ferreira SM, van Aarde RJ (2008) A rapid method to estimate population variables for African elephants. J Wildl Manage 72: 822–829.

[33] Jones ML (1993) Longevity of ungulates in captivity. Int Zoo Yearbook 32: 159–162.

[34] Eberhardt LL (1988) Using age structure data from changing populations. J Appl Ecol 25: 373–378.

[35] Edwards AWF (1972) Likelihood Cambridge: Cambridge University Press.

[36] Hood GM (2005) Poptools. Version 2.6.6. http://www.cse.csiro.au/poptools. Accessed 1 Jul 2005.

[37] Johnson JB, Omland KS (2004) Model selection in ecology and evolution. Trends Ecol Evol 19: 101–108. 16701236 10.1016/j.tree.2003.10.013

[38] Linklater WL, Swaisgood RR (2008) Reserve size, conspecific density, and translocation success for black rhinoceros. J Wildl Manage 7: 1059–1068.

[39] Greaver C, Ferreira SM, Slotow R (2014) Density-dependent regulation of the critically endangered black rhinoceros population in Ithala Game Reserve, South Africa. Austral Ecol 10.1111/aec.12101

[40] Linklater WL, Hutcheson IR (2010) Black rhinoceros are slow to colonize a harvested neighbour’s range. S Afr J Wildl Manage 40: 58–63.

[41] Schwabe F, Noack J, Goettert T, Starik N, Zeller U (2013) Investigating the spatial and temporal behavior of a translocated black rhino (*Diceros bicornis*) starter group on a private game farm in Namibia using camera traps and VHF radio telemetry. Arbeitsberichte des Geographisches Institut Humboldt-Universitat zu Berlin 175: 62–63.

[42] Eberhardt LL (2002) A paradigm for population analysis of long-lived vertebrates. Ecology 83: 2841–2854.

[43] Waldram MS, Bond WJ, Stock WD (2008) Ecological engineering by a mega-grazer: White Rhino impacts on a South African savanna. Ecosystems 11: 101–112.

[44] Owen-Smith N (1988) Megaherbivores: The influence of very large body size on ecology Cambridge: Cambridge University Press.

[45] Knight MH, Balfour D, Emslie RH (2013) Biodiversity management plan for the black rhinoceros (*Diceros bicornis*) in South Africa 2011–2020. Government Gazette South Africa 36096: 5–76.

[46] Rachlow JL, Berger J (1998) Reproduction and population density: trade-offs for the conservation of rhinos in situ. Anim Conserv 1: 101–106.

[47] Smit IPJ (2013) Systems approach towards surface water distribution in Kruger National Park, South Africa. Pachyderm 53: 91–98.

[48] Bennett AF, Haslem A, Cheal DC, Clarke MF, Jones RN, Koehn JD, et al (2009) Ecological processes: A key element in strategies for nature conservation. Ecol Manage Restor 10: 192–199.

[49] Humpreys J, Smith MLR 2014 The ‘rhinofication’ of South African security. International Affairs 90: 795–818.

[50] Milliken T, Shaw J (2012) The South Africa-Viet Nam rhino horn trade nexus: a deadly combination of institutional lapses, corrupt wildlife industry professionals and Asian crime syndicates Johannesburg: TRAFFIC.

[51] Ferreira SM, Okita-Ouma B (2012) A proposed framework for short-, medium- and long-term responses by range States to curb poaching for African rhino horns. Pachyderm 51: 60–74.

[52] Haas T, Ferreira SM (2015) Federated databases and actionable intelligence: Using social network analysis to disrupt transnational wildlife trafficking criminal networks. Security Informatics 10.1186/s13388-015-0018-8

[53] Child B (2012) The sustainable use approach could save South Africa's rhinos. S. Afr. J. Sci. 108: 21–25.

[54] Lunstrum E (2013) Green militarization: Anti-poaching efforts and the spatial contours of Kruger National Park. Annals of the Association of American Geographers, DOT: 10.1080/00045608.2014.912545

